# ANXIETY-DEPRESSIVE DISORDER IN A PATIENT WITH GRAVES’ DISEASE AND PSYCHOSOCIAL PROBLEMS

**DOI:** 10.1192/j.eurpsy.2023.1601

**Published:** 2023-07-19

**Authors:** M. Betriu, C. Becerra, S. Garcia, A. Vidal, E. Castan

**Affiliations:** 1 Subacute hospitalization unit, Hospital Sant Joan de Déu Lleida, Lleida; 2 CSMA Tarragona, Institut Pere Mata, Tarragona, Spain

## Abstract

**Introduction:**

There is clear evidence of the association of hypothyroidism with depression. It is known to be effective in some cases of adding triiodothyronine (T3) to antidepressant treatment in resistant depressive disorders. However, depression and anxiety can also be linked to hyperthyroidism.

Graves’ disease is an autoimmune disorder that is the most common cause of hyperthyroidism. Some of the symptoms associated with the disease are goiter, ophthalmopathy and psychiatric manifestations such as mood and anxiety disorders.

It is known that different psychosocial factors such as traumatic events, relevant life events, daily stressors, lack of social support, or different personality traits may correlate with Graves’ disease.

**Objectives:**

The case of an 18-year-old boy diagnosed with Mixed Adaptive Disorder and Graves’ Disease is presented.

**Methods:**

Clinical case presentation and non-systematic narrative review in PubMed.

**Results:**

Clinical case: 18-year-old male patient presenting with nervousness, obsessive thoughts, insomnia, decreased anorexia with marked weight loss, tachycardia, involuntary periorbital muscle movements, trichotillomania and wounds in the oral cavity secondary to bites in the context of serious problems with his family and with the law. Anxiolytic and antidepressant treatment is started but the paitent does not take regularly. Admission to Subacute Unit for clinical stabilization and containment of the situation at the social area. Through blood analysis, a diagnosis of Graves’ disease is made and antithyroid treatment is started, presenting significant clinical improvement. Later, with the adequate intake of the psychopharmacological treatment, aims a complete resolution of symptoms.

Review: 1)The association between anxious depressive symptoms and thyroid function is significant. 2) The psychiatric symptoms of Graves’ disease do not follow a specific pattern and are similar to those of an anxiety disorder or a primary anxiety-depressive disorder. 3)They have observed changes in psychopathological aspects in patients with subclinical hyperthyroidism. 4)In various studies it is shown that neuropsychiatric symptoms persist for a later time than thyroid function is normal and in some cases the complete resolution of these symptoms is not resolved. 5)Recent studies conclude that stress can be related to the debut and the evolution of Graves’ disorder despite the difficulty in quantifying it objectively.

**Conclusions:**

1) Routine screenings for thyroid disorders are important in patients with mood and anxiety disorders. 2) When neuropsychiatric symptoms persist despite normalization of thyroid function it should be considered the coexistence of a primary psychiatric disorder as well as the existence of psychosocial factors. 3) It is of interest to carry out research based on a biopsychosocial model to expand the study of the impact of stress on Graves’ Disease.

**Disclosure of Interest:**

None Declared

